# Geographic Variation in Maternal Smoking during Pregnancy in the Missouri Adolescent Female Twin Study (MOAFTS)

**DOI:** 10.1371/journal.pone.0153930

**Published:** 2016-04-21

**Authors:** Min Lian, Pamela A. Madden, Michael T. Lynskey, Graham A. Colditz, Christina N. Lessov-Schlaggar, Mario Schootman, Andrew C. Heath

**Affiliations:** 1 Department of Medicine, Washington University School of Medicine, St. Louis, MO, United States of America; 2 Cancer Prevention and Control Program, Alvin J. Siteman Cancer Center, Barnes-Jewish Hospital, and Washington University School of Medicine, St. Louis, MO, United States of America; 3 Department of Psychiatry, Washington University School of Medicine, St. Louis, MO, United States of America; 4 Addictions Department, Institute of Psychiatry, Psychology & Neuroscience, King’s College London, London, United Kingdom; 5 Department of Surgery, Washington University School of Medicine, St. Louis, MO, United States of America; 6 Department of Epidemiology, College for Public Health and Social Justice, St. Louis University, St. Louis, MO, United States of America; 7 Midwest Alcoholism Research Center, Washington University School of Medicine, St. Louis, MO, United States of America; University of Missouri, UNITED STATES

## Abstract

**Objective:**

Despite well-known adverse health effects of maternal smoking during pregnancy (MSP), it is still unclear if MSP varies geographically and if neighborhood socioeconomic deprivation (SED) plays an important role in MSP. This study aims to investigate small-area geographic variation in MSP and examine the association of SED with MSP.

**Methods:**

The Missouri Adolescent Female Twin Study (MOAFTS) is a cohort study of female like-sex twins born in Missouri to Missouri-resident parents during 1975–1985. Biological mothers completed a baseline interview in 1995–1998 and reported MSP with the twins. Residential address of the mother at birth was geocoded. We developed a census tract-level SED index using a common factor approach based on 21 area-level socioeconomic variables from the 1980 Census data. Multilevel logistic regressions estimated geographic heterogeneity (random effect) in MSP and the odds ratios (ORs, fixed effects) of neighborhood SED associated with MSP.

**Results:**

Of 1658 MOAFTS mothers, 35.2% reported any MSP and 21.9% reported MSP beyond the first trimester. Neighborhood SED was associated with any MSP (the highest vs. the lowest quartile: OR = 1.90, 95% confidence interval [CI] = 1.40–2.57, *P*_trend_<0.001) and MSP beyond the first trimester (OR = 1.98, 95% CI = 1.38–2.85, *P*_trend_ = 0.002) in unadjusted analyses. After adjusting for individual covariates (demographics, socioeconomic conditions, alcohol use, and parents’ cohabitation), neighborhood SED was not associated with MSP, but geographic variation still persisted in MSP (variance = 0.41, *P* = 0.003) and in MSP beyond the first trimester (variance = 0.82, *P*<0.001).

**Conclusions:**

Neighborhood SED was associated with MSP in unadjusted analyses but this association could be explained by individual socioeconomic conditions. Nonetheless, significant geographic variation in MSP persisted and was not accounted for by differences in neighborhood SED. To develop effective interventions to reduce MSP, further studies are necessary to explore underlying reasons for its geographic variation.

## Introduction

Cigarette smoking is an established risk factor for multiple chronic diseases and the leading preventable cause of mortality in the United States [1]. Excess mortality can be attributed to more than 21 common smoking-relevant diseases, including cancer, cardiovascular diseases, and respiratory diseases [2]. Tobacco control is the highest priority for advancing population health [3], which includes maternal smoking during pregnancy (MSP). In addition to hazards to pregnant women themselves, MSP could increase risks of adverse prenatal development [4], preterm birth [5], disruptive behaviors [6–9], socio-psychiatric disorders [10–13], obesity [14–16], and other diseases [17, 18] in their offspring. The U.S. CDC reported that the prevalence of MSP remained relatively stable between 10% and 15% during 2000–2011 in nine states included in the Pregnancy Risk Assessment Monitoring System (PRAMS) [19], while smoking in the general U.S. population has declined [20].

Previous studies have reported individual-level risk factors for MSP [21–31]. While some studies reported that MSP was associated with area-level characteristics [32–35], no previous study has assessed the extent of geographic variation in this behavior. It is also unclear if potential geographic variation in MSP can be attributed to some specific area-level characteristics, such as neighborhood socioeconomic deprivation and neighborhood tobacco environment. Such research could aid in the development of more effective public health interventions to reduce MSP. For example, public health officials could maximize the benefit of public health interventions by targeting specific geographic areas and reasons for elevated MSP prevalence in these areas.

In the current study, we applied a multilevel approach to examine the extent of geographic variation and the role of small-area socioeconomic deprivation (SED) in MSP using a population-representative sample of Missouri female twins.

## Materials and Methods

### Study Population

The Missouri Adolescent Female Twin Study (MOAFTS) is a prospective cohort study of female like-sex twins born in Missouri to Missouri-resident parents from July 1, 1975 through June 30, 1985. Monozygotic and dizygotic twins were ascertained through Missouri birth records. Study twins were recruited using a cohort-sequential design [36], and parent interviews were completed in 1995–1998. The twin cohort has been followed prospectively since median age 15, for about 20 years, through seven waves of data collection, to examine the development of their alcohol and tobacco use, other health behaviors and social/psychiatric disorders. In the current study, using the MOAFTS parent interview data (n = 1658), we investigated the extent of geographic variation and the role of neighborhood SED in MSP. The study, including the consent procedure, was approved by the Institutional Review Board at Washington University School of Medicine. All study participants provided their written informed consent to participant in this study.

### Study outcomes

Mothers who reported lifetime smoking of at least 100 cigarettes were classified as regular smokers. All regular smokers were asked 1) whether they had smoked during their pregnancy with the twins, including before they realized they were pregnant; and 2) for how many weeks or months they had smoked. We coded a) whether a mother had smoked during the first trimester, and b) whether she had smoked beyond the first trimester (which in most cases meant smoking throughout the pregnancy) to reflect the chronicity of MSP.

### Measures

#### Neighborhood socioeconomic deprivation (SED) index

The residential addresses from birth records were geocoded to obtain their coordinates and corresponding residential census tract using ArcGIS (version 10.2.2, ESRI Inc., Redland, CA). In all, 798 census tracts were included in the study sample, which study participants ranged from 1 to 9 (median: 2). To explore if geographic variation in MSP can be attributed to specific neighborhood characteristics, we assessed neighborhood socioeconomic deprivation, a commonly used neighborhood measure of area-level characteristic.

Since the twins were born during 1975–1985, we used 1980 Missouri Census data to develop a census tract-level statewide SED index. Similar to our previous study [37], we selected multiple socioeconomic and demographic variables in six domains, including 1) education (the percentage of population with less than high school education); 2) employment and occupation (the percentage of population unemployed, the percentage of population with working class); 3) housing conditions (the percentage of households without ownership, the percentage of households with vacancy, the percentage of households with > = 1 person per room, the percentage of households in poverty, the percentage of households female-headed with dependent children, the percentage of households with family income less than $30,000, the percentage of households with public assistance, the percentage of households without a car, the percentage of households without a phone, the percentage of households without plumbing, the percentage of households without a kitchen); 4) poverty and income (the percentage of population below the federal poverty line, income disparity); 5) racial composition (the percentage of non-Hispanic African American population, the percentage of Hispanic population); and 6) residential stability (the percentage of population aged 65 or above, the percentage of population living in the same residence in the past five years). We applied a principal component common factor analysis approach to examine the structure of these socioeconomic indicators, and selected seven variables that substantially contributed to the total variation of socioeconomic variables, including the percentage of population unemployed, the percentage of households with at least one person per room, the percentage of households female-headed with dependent children, the percentage of households with public assistance, the percentage of households without a car, the percentage of population below the federal poverty line, and the percentage of non-Hispanic African American population. These seven variables account for 40.5% of overall variation and have high internal consistency (Cronbach alpha = 0.92). After standardization and weighting by factor loading coefficients, these seven variables were summed as the final SED index. The SED index was categorized into quartiles based on the number of study participants in the census tracts in order to examine nonlinear trends in multilevel analysis. The highest SED quartile represents the most deprived category of neighborhood socioeconomic conditions.

#### Individual covariates

Three groups of individual characteristics during the pregnancy were considered as potential confounders to adjust the multilevel models, including 1) demographics (age and race), 2) individual socioeconomic conditions (mother’s education, father’s education, and family income), and 3) maternal alcohol use (alcohol drinking during pregnancy, and maternal history of alcohol dependence) and biological parents’ cohabitation. These individual characteristics were chosen based on previous literature. Mother’s age at twin birth was categorized into three groups: <18 years, 18–29 years, and >30 years. Race was dichotomized into White or African American. Both mother’s education and father’s education were categorized as ≤12 or >13 years. Family income was dichotomized at the cutoff of $45,000 per year based on the income distribution in the study population. The parents’ cohabitation was grouped as yes or no. Maternal alcohol dependence was assessed according to Diagnostic and Statistical Manual-IV (DSM-IV) criteria, using a telephone adaptation of the Semi-Structured Assessment for the Genetic of Alcoholism (SSAGA) diagnostic interview [38]. Alcohol dependence was defined as endorsing three or more of seven diagnostic criteria in the same 12-month period, lifetime. Both Alcohol use during pregnancy and maternal history of alcohol dependence were dichotomized as yes or no.

### Statistical analysis

Chi-square tests were used to examine potential differences in individual characteristics across quartiles of the neighborhood SED index. We applied a generalized linear mixed modeling approach to fit the multilevel logistic regression to quantify the geographic variation in and the association of neighborhood SED with MSP. The trend of SED association was tested by using the medians of each SED quartile in the models.

To examine if groups of individual covariates could explain potential geographic variation and the role of neighborhood SED, we fit four multivariate models adjusting for any one or all three groups of individual characteristics. Geographic variation in MSP was quantified using census tract-level variance from the fitted multilevel model. Since this variance has no meaningful unit and is hard to interpret by itself, we computed two heterogeneity measures, median odds ratio (MOR) and interquartile odds ratio (IqOR) to estimate the geographic heterogeneity in MSP based on commonly used odds ratios [39, 40]. Calculation of these two measures is described elsewhere [39, 40]. The fit of the multilevel models was based on the scaled deviance, with lower deviance indicating better fit [41]. The data management and statistical analysis were conducted in SAS System (version 9.3, SAS Institute Inc., Cary, North Carolina).

## Results

Of 1685 mothers with twins, 584 (35.2%) smoked during pregnancy, and 363 (21.9%) smoked beyond the first trimester of pregnancy. Mothers living in neighborhoods with higher SED were more likely to be younger, African American, with less education and lower family income, not cohabitating with the father of the twins, and with a personal history of alcohol dependence compared to those in neighborhoods with lower SED (Table 1).

**Table 1:** Characteristics of mothers of twins (N = 1658) from the Missouri Adolescent Female Twin Study (MOAFTS).

Variable	Neighborhood Socioeconomic Deprivation (SED) Quartile				*P* ^a^
	1st (Lowest SED) (n = 414)	2^nd^ (n = 422)	3^rd^ (n = 405)	4th (Highest SED) (n = 417)	
Smoking during pregnancy (MSP)					<0.001
Yes	111 (26.81)	152 (36.02)	152 (37.53)	169 (40.53)	
No	303 (73.19)	270 (63.98)	253 (62.47)	248 (59.47)	
MSP beyond 1^st^ trimester					0.002
Yes	64 (15.46)	92 (21.80)	102 (25.19)	105 (25.18)	
No	350 (84.54)	330 (78.20)	303 (74.81)	312 (74.82)	
Age at twin birth					<0.001
< 18 Years	5 (1.21)	12 (2.84)	19 (4.69)	27 (6.47)	
18–29 Years	276 (66.99)	324 (76.78)	320 (79.01)	323 (77.46)	
30+ Years	131 (31.80)	86 (20.38)	66 (16.30)	67 (16.07)	
Missing	2	0	0	0	
Race					<0.001
White	408 (98.55)	403 (95.50)	388 (95.80)	249 (59.71)	
AA	6 (1.45)	19 (4.50)	17 (4.20)	168 (40.29)	
Education (Mom)					<0.001
< = 12 years	15 (3.62)	46 (10.90)	47 (11.60)	71 (17.03)	
> = 13 years	348 (84.06)	311 (73.70)	295 (72.84)	267 (64.03)	
Unknown	51 (12.32)	65 (15.40)	63 (15.56)	79 (18.94)	
Education (Dad)					<0.001
< = 12 years	15 (3.62)	33 (7.82)	49 (12.10)	61 (14.63)	
> = 13 years	345 (83.33)	322 (76.30)	280 (69.14)	249 (59.71)	
Unknown	54 (13.04)	67 (15.88)	76 (18.77)	107 (25.66)	
Household income					<0.001
< = $45,000	168 (40.58)	247 (58.53)	253 (62.47)	324 (77.70)	
> $45,000	233 (56.28)	167 (39.57)	142 (35.06)	89 (21.34)	
Unknown	13 (3.14)	8 (1.90)	10 (2.47)	4 (0.96)	
Parents’ cohabitation					<0.001
Yes	358 (86.47)	353 (83.65)	327 (80.74)	274 (65.71)	
No	7 (1.69)	19 (4.50)	22 (5.43)	70 (16.79)	
Missing	49 (11.84)	50 (11.85)	56 (13.83)	73 (17.51)	
Alcohol use during pregnancy					0.211
Yes	148 (35.75)	125 (29.62)	126 (31.11)	114 (27.34)	
No	264 (63.77)	293 (69.43)	276 (68.15)	298 (71.46)	
Unknown	2 (0.48)	4 (0.95)	3 (0.74)	5 (1.20)	
Maternal history of alcohol dependence					0.023
Yes	12 (2.90)	16 (3.79)	26 (6.42)	28 (6.71)	
No	402 (97.10)	406 (96.21)	379 (93.58)	389 (93.29)	

^a^ Chi-square test.

### MSP

The prevalence of MSP was significantly higher for mothers residing in neighborhoods with the highest SED quartile than those in neighborhoods with the lowest SED quartile (40.5% vs. 26.8%, *P* < 0.001) (Table 1). Mothers who lived in neighborhoods with the highest SED quartile were more likely to smoke during pregnancy (Table 2 Model 1: odds ratio [OR] = 1.90, 95% confidence interval [CI] = 1.40–2.57, *P*_trend_<0.001) compared with those who lived in neighborhoods with the lowest SED quartile. The associations remained after adjusting for age and race (Model 2) or for parents’ cohabitation and maternal alcohol use (Model 4), but not after adjusting for individual socioeconomic conditions (Model 3) or for all three groups of individual covariates (Model 5). However, considerable geographic variation in MSP remained in Model 5 (MOR = 1.84, IqOR = 4.37). If two mothers with the same individual characteristics were randomly selected from two different neighborhoods, their likelihoods of MSP were 1.84 times different (on average). Meanwhile, a mother, randomly selected from the first quartile of participants living in neighborhoods with the highest likelihood of MSP, was 4.37 times more likely to smoke during pregnancy than a mother randomly selected from the fourth quartile of participants living in neighborhoods with the lowest likelihood of MSP. Model 5 showed that MSP was associated with several individual characteristics, including less education, lower family income, any alcohol use during pregnancy, and maternal history of alcohol dependence (Table 3).

**Table 2:** Geographic variation in and the association of neighborhood socioeconomic deprivation with maternal smoking during pregnancy (MSP) (MOR, median odds ratio; IqOR, interquartile odds ratio). Model 1 was not adjusted for any individual characteristics; Model 2 was adjusted for age and race; Model 3 was adjusted for individual socioeconomic conditions (mom’s education, dad’s education, and household income); Model 4 was adjusted for biological parents’ cohabitation and alcohol use (maternal alcohol use during pregnancy, and maternal history of alcohol dependence); Model 5 was adjusted for all individual covariates.

	Model 1	Model 2	Model 3	Model 4	Model 5
**MSP**					
**Fixed effects**	**OR (95% CI)**	**OR (95% CI)**	**OR (95% CI)**	**OR (95% CI)**	**OR (95% CI)**
Socioeconomic Deprivation					
1st (least)	1.00	1.00	1.00	1.00	1.00
2^nd^	1.54 (1.12–2.11)	1.52 (1.10–2.08)	1.26 (0.91–1.76)	1.63 (1.18–2.25)	1.31 (0.93–1.85)
3^rd^	1.65 (1.21–2.25)	1.62 (1.18–2.21)	1.24 (0.89–1.72)	1.67 (1.21–2.29)	1.26 (0.89–1.77)
4^th^ (most)	1.90 (1.40–2.57)	1.87 (1.34–2.63)	1.18 (0.85–1.65)	1.88 (1.37–2.60)	1.30 (0.90–1.89)
P for trend	<0.001	0.002	0.621	0.002	0.317
**Random effects**	**Index**	**Index**	**Index**	**Index**	**Index**
Variance	0.26 (P = 0.021)	0.25 (P = 0.023)	0.36 (P = 0.005)	0.27 (P = 0.021)	0.41 (P = 0.003)
MOR	1.62	1.61	1.78	1.65	1.84
IqOR	3.21	3.18	3.99	3.33	4.37
Scaled Deviance	2043	2038	1937	1973	1863
**MSP beyond 1**^**st**^ **trimester**					
**Fixed effects**	**OR (95% CI)**	**OR (95% CI)**	**OR (95% CI)**	**OR (95% CI)**	**OR (95% CI)**
Socioeconomic Deprivation					
1st	1.00	1.00	1.00	1.00	1.00
2nd	1.54 (1.05–2.25)	1.56 (1.07–2.28)	1.22 (0.82–1.82)	1.56 (1.07–2.28)	1.26 (0.84–1.88)
3rd	1.98 (1.37–2.85)	2.00 (1.38–2.91)	1.46 (0.99–2.16)	1.91 (1.32–2.76)	1.49 (1.00–2.20)
4th	1.98 (1.38–2.85)	2.22 (1.49–3.29)	1.18 (0.79–1.75)	1.85 (1.27–2.70)	1.40 (0.91–2.15)
P for trend	0.002	<0.001	0.758	0.009	0.202
** Random effects**	**Index**	**Index**	**Index**	**Index**	**Index**
Variance	0.65 (P<0.001)	0.66 (P<0.001)	0.89 (P<0.001)	0.64 (P<0.001)	0.82 (P<0.001)
MOR	2.16	2.17	2.45	2.14	2.37
IqOR	6.40	6.46	8.71	6.28	8.03
Scaled Deviance	1745	1737	1673	1713	1629

**Table 3:** Associations of individual maternal characteristics with maternal smoking during pregnancy (MSP). Both models were adjusted for neighborhood socioeconomic deprivation.

Variable	OR (95% CI)	
	MSP	MSP beyond 1st trimester
Age at twin birth		
< 18 Years	0.71 (0.39–1.29)	0.82 (0.44–1.53)
18–29 Years	1.00	1.00
30+ Years	0.98 (0.75–1.29)	1.25 (0.93–1.68)
Race		
White	1.00	1.00
AA	0.82 (0.56–1.22)	0.64 (0.41–0.99)
Education (Mom)		
< = 12 years	3.16 (2.19–4.56)	2.84 (1.98–4.08)
> = 13 years	1.00	1.00
Education (Dad)		
< = 12 years	1.96 (1.34–2.88)	1.85 (1.26–2.71)
> = 13 years	1.00	1.00
Household income		
< = $45,000	2.07 (1.61–2.65)	2.37 (1.79–3.14)
> $45,000	1.00	1.00
Parents’ cohabitation		
Yes	1.00	1.00
No	1.13 (0.71–1.80)	1.25 (0.77–2.04)
Alcohol use during pregnancy		
Yes	2.59 (2.04–3.27)	1.61 (1.24–2.07)
No	1.00	1.00
Maternal history of alcohol dependence		
Yes	2.15 (1.30–3.55)	2.58 (1.59–4.17)
No	1.00	1.00

### MSP beyond the first trimester

Results were similar for MSP beyond the first trimester. The prevalence of continued MSP was significantly higher for mothers residing in neighborhoods with the highest SED quartile than those in neighborhoods with the lowest SED quartile (25.2% vs. 15.5%, *P* = 0.002) (Table 1). Neighborhood SED was significantly associated with continued MSP (Table 2 Model 1, the most vs. the least deprived: OR = 1.98, 95% CI = 1.38–2.85, *P*_trend_ = 0.002). The association was not changed after adjusting for demographics (Model 2) or maternal alcohol use and parents’ cohabitation (Model 4), but became nonsignificant after adjusting for individual socioeconomic conditions in Model 3 (*P*_trend_ = 0.758) or all individual covariates (Model 5, the most vs. the least deprived quartile: OR = 1.40, 95% CI = 0.91–2.15, *P*_trend_ = 0.202). However, considerable geographic variation remained for MSP beyond the first trimester in Model 5 (MOR = 2.37, IqOR = 8.03). The individual characteristics associated with MSP beyond the first trimester included less education, lower family income, any alcohol use during pregnancy, and maternal history of alcohol dependence (Table 3).

## Discussion

The prevalence of MSP still remains high in the United States, although its adverse health effects have been well-understood [4–18]. The U.S. CDC reported that the prevalence of MSP decreased from 18.4% to 11.4% during 1990–2002 [42]. However, the prevalence of MSP was significantly higher in Missouri (24.8% in 1990) than in the U.S. Although slightly decreased, the prevalence of MSP remained high in 2002 (18.2%) [42] and remained about stable (15–20%) from 2000–2011 in Missouri [19]. As one of three national objectives relevant to MSP, the U.S. *Healthy People* 2020 goal (HP 2020) aims to reduce the prevalence rate of MSP to 1.4%.[43] Current rates of MSP are far higher than this goal. There is therefore an urgent need to design and implement more effective public health strategies and policies to achieve this objective. Examining the extent of geographic variation in MSP is one key aspect.

The main purpose of our study was to examine geographic variation in and the association of neighborhood SED with MSP. In the female twin cohort, with uniquely rich data on geographic information and exposures during pregnancy of the mothers of the twins, we observed that MSP varied geographically and that neighborhood SED was associated with MSP in unadjusted models. However, individual socioeconomic conditions explained the relationship between higher neighborhood SED and increased likelihood of MSP. Nonetheless, the measured variables included in our study did not explain the substantial geographic variation in MSP. Similar results were found in continued MSP.

Some studies have investigated individual factors associated with MSP, including older age of mother’s first pregnancy [21, 22], low socioeconomic status [21, 22], being unmarried [23], lower educational attainment [24–26], mental health problems [27–29], irregular doctor checkup [21, 22], limited or no religious participation [26, 30], and limited social support [31]. Area-level studies have reported that MSP was associated with higher neighborhood social capital [33], lower area-level education [34], more residential segregation [35], absence of local indoor smoking ordinance [44], and lower state-level cigarette tax rates [45]. No previous study has, to our knowledge, investigated the extent of geographic variation in MSP. In the current study, we found that the neighborhood SED association could be explained by individual socioeconomic status. In other words, mothers in the most deprived neighborhoods were more likely to smoke because they were themselves of lower socioeconomic status.

Both neighborhood- and individual-level socioeconomic conditions did not account for geographic variation in MSP. One potential explanation may be geographic access to tobacco outlets. Previous studies reported that greater concentration of and shorter travel distance to tobacco retailers were associated with higher likelihood of smoking among the general adult population [46, 47]. Better understanding of geographic patterns in MSP and underlying reasons will facilitate prioritizing limited health and intervention resources to neighborhoods with the highest rates of MSP.

Some studies reported the effectiveness of intervention efforts aiming to reduce MSP. Pharmaceutical approaches, such as nicotine replacement therapy, were reported to have limited effects on increasing smoking cessation during pregnancy [48]. Recently, a randomized controlled trial found that a physical activity intervention did not improve smoking cessation during pregnancy [49]. Randomized trials indicated that a 5As-based brief intervention successfully increased smoking cessation during pregnancy [50, 51], while a computer-assisted, simplified, and low-intensity contingency management (CM-Lite) intervention was not effective [50]. Two phase II randomized controlled trials showed that financial incentives could increase smoking cessation during pregnancy [52, 53]. A meta-analysis included 12 trials showed that self-help interventions were more effective in smoking cessation compared to standard care during pregnancy [54]. It was found that physician training and individual counseling was useful in reducing MSP [55]. Similar to the general population, pregnant women were responsive to higher price and taxes of cigarette [56]. These intervention studies did not take into account the impact of geographic heterogeneity in MSP on the effectiveness of the intervention efforts. To control tobacco use and reduce disparities in smoking during pregnancy in a more fruitful way, future tobacco control strategies should be designed in a multilevel framework, including strengthening health communication among primary care providers and OB/GYN physicians and women during pregnancy, geographically restricting tobacco retail outlets, or increasing local cigarette tax, especially in geographic areas with higher risk of MSP.

Our study has strengths and limitations. In the first study of this kind, we quantified geographic variation in MSP and attempted to explore the potential reasons. The recruitment of the MOAFTS cohort was based on official birth records, and we also verified that there was no significant difference between the MOAFTS twin births and entire female births in terms of socioeconomic environment in the state of Missouri in the same birth years. Therefore, our study sample was largely representative of all female births in Missouri. We applied a multivariable approach to develop a composite neighborhood SED index which could capture broader deprivation concepts compared to a single socioeconomic indicator [57]. Although the inaccuracy of residential address might also introduce geocoding bias and misclassification of neighborhood SED, official birth records are less likely subject to this issue. Since the parent interview was conducted after the twin births, it is possible that the information about MSP might have suffered from recall bias. Our previous study showed that mothers are accurate reporters of their MSP based on their twin sister’s reports [58]. Prospective observation may be needed to assess longitudinal consistency of self-reported MSP in the future. In addition, due to unavailability of historic information about other neighborhood variables, such as tobacco outlets distribution at the time of the twins’ births, we can only use census data to describe neighborhood characteristics. Our analysis of historic data in the MOAFTS cohort may not directly benefit current tobacco control efforts. However, our findings strongly suggest that further studies are needed to investigate the relationship of geographic variation in MSP and neighborhood characteristics other than socioeconomic deprivation. The current findings will also be helpful for us to further investigate the impact of geographic heterogeneity in MSP on the development of smoking behaviors among their children in the MOAFTS cohort.

In conclusion, significant geographic variation in MSP existed in the MOAFTS cohort. Neighborhood SED did not fully account for this geographic variation in MSP. Further studies are necessary to replicate our findings and identify potential mechanisms underlying the observed geographic clustering of tobacco use during pregnancy. This will help public health professionals implement evidence-based public health interventions to reduce disparities in MSP.

## References

[1] Mokdad AH, Marks JS, Stroup DF, Gerberding JL. Actual causes of death in the United States, 2000. JAMA. 2004;291:1238–45. 15010446 10.1001/jama.291.10.1238

[2] Carter BD, Abnet CC, Feskanich D, Freedman ND, Hartge P, Lewis CE, et al Smoking and mortality—beyond established causes. N Engl J Med. 2015;372(7):631–40. Epub 2015/02/12. 10.1056/NEJMsa1407211 . 25671255

[3] Colditz GA. Smoke alarm—tobacco control remains paramount. N Engl J Med. 2015;372(7):665–6. Epub 2015/02/12. 10.1056/NEJMe1414318 . 25671261

[4] Lee LJ, Lupo PJ. Maternal smoking during pregnancy and the risk of congenital heart defects in offspring: a systematic review and metaanalysis. Pediatr Cardiol. 2013;34(2):398–407. Epub 2012/08/14. 10.1007/s00246-012-0470-x . 22886364

[5] Ko TJ, Tsai LY, Chu LC, Yeh SJ, Leung C, Chen CY, et al Parental smoking during pregnancy and its association with low birth weight, small for gestational age, and preterm birth offspring: a birth cohort study. Pediatr Neonatol. 2014;55(1):20–7. Epub 2013/07/16. 10.1016/j.pedneo.2013.05.005 . 23850094

[6] Wakschlag LS, Pickett KE, Cook E Jr., Benowitz NL, Leventhal BL. Maternal smoking during pregnancy and severe antisocial behavior in offspring: a review. Am J Public Health. 2002;92(6):966–74. Epub 2002/05/31. 12036791 10.2105/ajph.92.6.966 PMC1447496

[7] Al Mamun A, O'Callaghan FV, Alati R, O'Callaghan M, Najman JM, Williams GM, et al Does maternal smoking during pregnancy predict the smoking patterns of young adult offspring? A birth cohort study. Tob Control. 2006;15(6):452–7. Epub 2006/11/30. 10.1136/tc.2006.016790 17130374 PMC2563674

[8] Rydell M, Magnusson C, Cnattingius S, Granath F, Svensson AC, Galanti MR. Exposure to maternal smoking during pregnancy as a risk factor for tobacco use in adult offspring. Am J Epidemiol. 2014;179(12):1409–17. Epub 2014/04/25. 10.1093/aje/kwu074 . 24761008

[9] Taylor AE, Howe LD, Heron JE, Ware JJ, Hickman M, Munafo MR. Maternal smoking during pregnancy and offspring smoking initiation: assessing the role of intrauterine exposure. Addiction. 2014;109(6):1013–21. Epub 2014/02/14. 10.1111/add.12514 24521169 PMC4114534

[10] Obel C, Olsen J, Henriksen TB, Rodriguez A, Jarvelin MR, Moilanen I, et al Is maternal smoking during pregnancy a risk factor for hyperkinetic disorder?—Findings from a sibling design. Int J Epidemiol. 2011;40(2):338–45. Epub 2010/11/16. 10.1093/ije/dyq185 . 21075808

[11] Langley K, Heron J, Smith GD, Thapar A. Maternal and paternal smoking during pregnancy and risk of ADHD symptoms in offspring: testing for intrauterine effects. Am J Epidemiol. 2012;176(3):261–8. Epub 2012/07/14. 10.1093/aje/kwr510 22791738 PMC3406617

[12] Talati A, Bao Y, Kaufman J, Shen L, Schaefer CA, Brown AS. Maternal smoking during pregnancy and bipolar disorder in offspring. Am J Psychiatry. 2013;170(10):1178–85. Epub 2013/10/03. 10.1176/appi.ajp.2013.12121500 24084820 PMC4086419

[13] Shenassa ED, Papandonatos GD, Rogers ML, Buka SL. Elevated Risk of Nicotine Dependence Among Sib-pairs Discordant for Maternal Smoking During Pregnancy: Evidence from a 40-year Longitudinal Study. Epidemiology. 2015;26(3):441–7. Epub 2015/03/15. . 25767988 10.1097/EDE.0000000000000270 PMC4455876

[14] Durmus B, Kruithof CJ, Gillman MH, Willemsen SP, Hofman A, Raat H, et al Parental smoking during pregnancy, early growth, and risk of obesity in preschool children: the Generation R Study. Am J Clin Nutr. 2011;94(1):164–71. Epub 2011/05/20. 10.3945/ajcn.110.009225 . 21593510

[15] Howe LD, Matijasevich A, Tilling K, Brion MJ, Leary SD, Smith GD, et al Maternal smoking during pregnancy and offspring trajectories of height and adiposity: comparing maternal and paternal associations. Int J Epidemiol. 2012;41(3):722–32. Epub 2012/03/13. 10.1093/ije/dys025 22407859 PMC3396309

[16] Ino T, Shibuya T, Saito K, Inaba Y. Relationship between body mass index of offspring and maternal smoking during pregnancy. Int J Obes (Lond). 2012;36(4):554–8. Epub 2011/12/21. 10.1038/ijo.2011.255 . 22184058

[17] Dior UP, Lawrence GM, Sitlani C, Enquobahrie D, Manor O, Siscovick DS, et al Parental smoking during pregnancy and offspring cardio-metabolic risk factors at ages 17 and 32. Atherosclerosis. 2014;235(2):430–7. Epub 2014/06/18. 10.1016/j.atherosclerosis.2014.05.937 24937467 PMC4123626

[18] Mattsson K, Jonsson I, Malmqvist E, Larsson HE, Rylander L. Maternal smoking during pregnancy and offspring type 1 diabetes mellitus risk: accounting for HLA haplotype. Eur J Epidemiol. 2015;30(3):231–8. Epub 2015/01/13. 10.1007/s10654-014-9985-1 . 25576078

[19] Centers for Disease Control and Prevention. Information for Health Care Providers and Public Health Professionals: Preventing Tobacco Use During Pregnancy. Available from: http://www.cdc.gov/reproductivehealth/maternalinfanthealth/tobaccousepregnancy/providers.html.

[20] Jamal A, Agaku IT, O'Connor E, King BA, Kenemer JB, Neff L. Current cigarette smoking among adults—United States, 2005–2013. MMWR Morb Mortal Wkly Rep. 2014;63(47):1108–12. Epub 2014/11/27. . 25426653 PMC5779518

[21] Al-Sahab B, Saqib M, Hauser G, Tamim H. Prevalence of smoking during pregnancy and associated risk factors among Canadian women: a national survey. BMC Pregnancy Childbirth. 2010;10:24 Epub 2010/05/26. 10.1186/1471-2393-10-24 20497553 PMC2885995

[22] Cui Y, Shooshtari S, Forget EL, Clara I, Cheung KF. Smoking during pregnancy: findings from the 2009–2010 Canadian Community Health Survey. PLoS One. 2014;9(1):e84640 Epub 2014/01/15. 10.1371/journal.pone.0084640 24416257 PMC3885577

[23] Kiernan K, Pickett KE. Marital status disparities in maternal smoking during pregnancy, breastfeeding and maternal depression. Soc Sci Med. 2006;63(2):335–46. Epub 2006/02/14. 10.1016/j.socscimed.2006.01.006 . 16472900

[24] Gilman SE, Breslau J, Subramanian SV, Hitsman B, Koenen KC. Social factors, psychopathology, and maternal smoking during pregnancy. Am J Public Health. 2008;98(3):448–53. Epub 2007/06/30. 10.2105/ajph.2006.102772 17600245 PMC2253564

[25] Higgins ST, Heil SH, Badger GJ, Skelly JM, Solomon LJ, Bernstein IM. Educational disadvantage and cigarette smoking during pregnancy. Drug Alcohol Depend. 2009;104 Suppl 1:S100–5. Epub 2009/05/16. 10.1016/j.drugalcdep.2009.03.013 19442460 PMC2763386

[26] Baron R, Mannien J, de Jonge A, Heymans MW, Klomp T, Hutton EK, et al Socio-demographic and lifestyle-related characteristics associated with self-reported any, daily and occasional smoking during pregnancy. PLoS One. 2013;8(9):e74197 Epub 2013/09/11. 10.1371/journal.pone.0074197 24019956 PMC3760841

[27] Zhu SH, Valbo A. Depression and smoking during pregnancy. Addict Behav. 2002;27(4):649–58. Epub 2002/08/22. . 12188598 10.1016/s0306-4603(01)00199-x

[28] Eiden RD, Leonard KE, Colder CR, Homish GG, Schuetze P, Gray TR, et al Anger, hostility, and aggression as predictors of persistent smoking during pregnancy. J Stud Alcohol Drugs. 2011;72(6):926–32. Epub 2011/11/05. 22051206 10.15288/jsad.2011.72.926 PMC3211963

[29] Kratz LM, Vaughan EL. Mental health problems, legal involvement, and smoking during pregnancy. Subst Use Misuse. 2012;47(6):718–25. Epub 2012/03/14. 10.3109/10826084.2012.664238 . 22409667

[30] Gillum RF, Sullins DP. Cigarette smoking during pregnancy: independent associations with religious participation. South Med J. 2008;101(7):686–92. Epub 2008/06/27. 10.1097/SMJ.0b013e31817a76cc . 18580727

[31] Masho SW, Do E, Adekoya S. Social Support and Smoking during Pregnancy. J Womens Health Care. 2014;3 Epub 2015/01/17. 10.4172/2167-0420.1000179 25593789 PMC4292848

[32] Bell JF, Zimmerman FJ, Mayer JD, Almgren GR, Huebner CE. Associations between residential segregation and smoking during pregnancy among urban African-American women. J Urban Health. 2007;84(3):372–88. Epub 2007/01/18. 10.1007/s11524-006-9152-4 17226080 PMC2231827

[33] Shoff C, Yang TC. Understanding maternal smoking during pregnancy: does residential context matter? Soc Sci Med. 2013;78:50–60. Epub 2012/12/19. 10.1016/j.socscimed.2012.11.027 23246395 PMC3545052

[34] Raisanen S, Kramer MR, Gissler M, Saari J, Hakulinen-Viitanen T, Heinonen S. Smoking during pregnancy was up to 70% more common in the most deprived municipalities—a multilevel analysis of all singleton births during 2005–2010 in Finland. Prev Med. 2014;67:6–11. Epub 2014/07/02. 10.1016/j.ypmed.2014.06.026 . 24983887

[35] Yang TC, Shoff C, Noah AJ, Black N, Sparks CS. Racial segregation and maternal smoking during pregnancy: a multilevel analysis using the racial segregation interaction index. Soc Sci Med. 2014;107:26–36. Epub 2014/03/08. 10.1016/j.socscimed.2014.01.030 24602968 PMC4029363

[36] Heath AC, Madden PA, Grant JD, McLaughlin TL, Todorov AA, Bucholz KK. Resiliency factors protecting against teenage alcohol use and smoking: influences of religion, religious involvement and values, and ethnicity in the Missouri Adolescent Female Twin Study. Twin Res. 1999;2(2):145–55. Epub 1999/09/10. . 10480749 10.1375/136905299320566013

[37] Lian M, Schootman M, Doubeni CA, Park Y, Major JM, Torres Stone RA, et al Geographic Variation in Colorectal Cancer Survival and the Role of Small-Area Socioeconomic Deprivation: A Multilevel Survival Analysis of the NIH-AARP Diet and Health Study Cohort. Am J Epidemiol. 2011;174(7):828–38. 10.1093/aje/kwr162 21836166 PMC3203377

[38] Bucholz KK, Cadoret R, Cloninger CR, Dinwiddie SH, Hesselbrock VM, Nurnberger JI Jr., et al A new, semi-structured psychiatric interview for use in genetic linkage studies: a report on the reliability of the SSAGA. J Stud Alcohol. 1994;55(2):149–58. Epub 1994/03/01. . 8189735 10.15288/jsa.1994.55.149

[39] Larsen K, Merlo J. Appropriate assessment of neighborhood effects on individual health: integrating random and fixed effects in multilevel logistic regression. Am J Epidemiol. 2005;161(1):81–8. . 15615918 10.1093/aje/kwi017

[40] Chaix B, Merlo J, Subramanian SV, Lynch J, Chauvin P. Comparison of a spatial perspective with the multilevel analytical approach in neighborhood studies: the case of mental and behavioral disorders due to psychoactive substance use in Malmo, Sweden, 2001. Am J Epidemiol. 2005;162(2):171–82. . 15972939 10.1093/aje/kwi175

[41] McCullagh P, Nelder JA. Generalized Linear Models. Second Edition ed: London: Chapman and Hall; 1989.

[42] Centers for Disease Control and Prevention. Smoking during pregnancy—United States, 1990–2002. MMWR Morb Mortal Wkly Rep. 2004;53(39):911–5. Epub 2004/10/08. . 15470322

[43] Healthy People 2020. www.healthypeople.gov/2020/.

[44] Nguyen KH, Wright RJ, Sorensen G, Subramanian SV. Association between local indoor smoking ordinances in Massachusetts and cigarette smoking during pregnancy: a multilevel analysis. Tob Control. 2013;22(3):184–9. Epub 2011/12/15. 10.1136/tobaccocontrol-2011-050157 22166267 PMC3401240

[45] Hawkins SS, Baum CF. Impact of state cigarette taxes on disparities in maternal smoking during pregnancy. Am J Public Health. 2014;104(8):1464–70. Epub 2014/06/13. 10.2105/ajph.2014.301955 24922149 PMC4103241

[46] Chuang YC, Cubbin C, Ahn D, Winkleby MA. Effects of neighbourhood socioeconomic status and convenience store concentration on individual level smoking. J Epidemiol Community Health. 2005;59:568–73. 15965140 10.1136/jech.2004.029041 PMC1757087

[47] Pearce J, Hiscock R, Moon G, Barnett R. The neighbourhood effects of geographical access to tobacco retailers on individual smoking behaviour. J Epidemiol Community Health. 2009;63(1):69–77. 10.1136/jech.2007.070656 18628269

[48] Coleman T, Chamberlain C, Davey MA, Cooper SE, Leonardi-Bee J. Pharmacological interventions for promoting smoking cessation during pregnancy. The Cochrane database of systematic reviews. 2015;12:Cd010078 Epub 2015/12/23. 10.1002/14651858.CD010078.pub2 . 26690977

[49] Ussher M, Lewis S, Aveyard P, Manyonda I, West R, Lewis B, et al Physical activity for smoking cessation in pregnancy: randomised controlled trial. Bmj. 2015;350:h2145 Epub 2015/05/16. 10.1136/bmj.h2145 ; PubMed Central PMCID: PMCPmc4431606. 25976288 PMC4431606

[50] Ondersma SJ, Svikis DS, Lam PK, Connors-Burge VS, Ledgerwood DM, Hopper JA. A randomized trial of computer-delivered brief intervention and low-intensity contingency management for smoking during pregnancy. Nicotine Tob Res. 2012;14(3):351–60. Epub 2011/12/14. 10.1093/ntr/ntr221 22157229 PMC3281243

[51] Bailey BA. Effectiveness of a Pregnancy Smoking Intervention: The Tennessee Intervention for Pregnant Smokers Program. Health education & behavior: the official publication of the Society for Public Health Education. 2015;42(6):824–31. Epub 2015/07/15. . 26157040 10.1177/1090198115590780

[52] Tappin D, Bauld L, Purves D, Boyd K, Sinclair L, MacAskill S, et al Financial incentives for smoking cessation in pregnancy: randomised controlled trial. Bmj. 2015;350:h134 Epub 2015/01/30. 10.1136/bmj.h134 . 25627664

[53] Boyd KA, Briggs AH, Bauld L, Sinclair L, Tappin D. Are financial incentives cost-effective to support smoking cessation during pregnancy? Addiction. 2016;111(2):360–70. Epub 2015/09/16. 10.1111/add.13160 . 26370095

[54] Naughton F, Prevost AT, Sutton S. Self-help smoking cessation interventions in pregnancy: a systematic review and meta-analysis. Addiction. 2008;103(4):566–79. Epub 2008/03/15. 10.1111/j.1360-0443.2008.02140.x . 18339103

[55] Secker-Walker RH, Solomon LJ, Flynn BS, Skelly JM, Mead PB. Reducing smoking during pregnancy and postpartum: physician's advice supported by individual counseling. Prev Med. 1998;27(3):422–30. Epub 1998/06/05. 10.1006/pmed.1998.0287 . 9612832

[56] Ringel JS, Evans WN. Cigarette taxes and smoking during pregnancy. Am J Public Health. 2001;91(11):1851–6. Epub 2001/10/31. 11684615 10.2105/ajph.91.11.1851 PMC1446890

[57] Lian M, Perez M, Liu Y, Schootman M, Frisse A, Foldes E, et al Neighborhood socioeconomic deprivation, tumor subtypes, and causes of death after non-metastatic invasive breast cancer diagnosis: a multilevel competing-risk analysis. Breast Cancer Res Treat. 2014;147(3):661–70. Epub 2014/09/23. 10.1007/s10549-014-3135-z 25234843 PMC4181526

[58] Heath AC, Knopik VS, Madden PA, Neuman RJ, Lynskey MJ, Slutske WS, et al Accuracy of mothers' retrospective reports of smoking during pregnancy: comparison with twin sister informant ratings. Twin Res. 2003;6(4):297–301. Epub 2003/09/27. 10.1375/136905203322296656 . 14511436

